# Attitudes to knee osteoarthritis and total knee replacement in Arab women: a qualitative study

**DOI:** 10.1186/1756-0500-6-406

**Published:** 2013-10-10

**Authors:** Abdullah Al-Taiar, Reem Al-Sabah, Ehab Elsalawy, Dia Shehab, Shaima Al-Mahmoud

**Affiliations:** 1 Department of Community Medicine and Behavioural Sciences, Faculty of Medicine, Kuwait University, Box: 24923, Safat 13110, Kuwait; 2 Al-Razi Hospital, Kuwait, Kuwait; 3 Department of Medicine, Faculty of Medicine, Kuwait University, Box: 24923, Safat 13110, Kuwait; 4 Department of Psychology, College of Arts and Sciences, Kent State University, Kent, USA

**Keywords:** Knee, Pain, Kuwait, Arthroplasty, Middle East

## Abstract

**Background:**

Total Knee Arthroplasty (TKA) is offered to patients with knee osteoarthritis (OA) in the oil-rich countries in the Gulf region without adequate understanding of their perceptions, preferences or pain experiences. This study aimed to explore the pain experience and mobility limitation as well as the patient’s decision making process to undertake TKA among women with knee pain in the waiting list for surgery.

**Methods:**

Five focus group discussions were conducted comprised of 39 women with severe knee OA from the waiting list for TKA in the only orthopaedic hospital in Kuwait. Discussions were recorded, transcribed and coded for themes to identify the factors considered to be important in decision-making for TKA.

**Results:**

Experiencing knee pain was central to daily living and affected patients and their families. Mobility limitation was shaped by a strong sense of expected obligation to take care of the family. Two major sources of TKA delay were identified; one was due to late clinical advice to undergo TKA which was the result of receiving several consultations from different clinicians each of whom tried the medical management for OA. The second delay occurred after the clinical advice for TKA and was mainly due to ambivalence of patients because of fear of the operation and the lack of information about TKA that resulted in unclear expectations of the surgery.

**Conclusions:**

Both verbal and written information about TKA should be provided as part of preoperative rehabilitation. This is critical to improve doctor-patient interactions and facilitate informed decision about the procedure and thus achieve patient-centered healthcare.

## Background

Knee pain is a major public health problem that causes considerable disability and impact on quality of life, particularly among elderly people. Most cases occur as a result of osteoarthritis (OA), a disease that is diagnosed mostly on clinical ground and is more common among women [1-3]. It is estimated that 25% of those who are 50 years or over suffer from chronic knee pain in the UK [4] while more than 29% of people who are 65 years or above have knee pain in Italy [5,6]. Some studies have estimated the prevalence of knee OA to be 44% among people who are 80 years of age or older [1]. In Kuwait, the age adjusted prevalence of musculoskeletal pain was estimated to be 35.7% among females and 20.2% among males with knee being one of the most common sites [7]. Knee OA is set to increase in Kuwait and other countries in the Gulf region because of ageing populations and ever increasing prevalence of obesity.

Currently there is no evidence-based medical treatment to stop or reverse the progression of knee OA and thus the available treatments focus on managing the pain and disability. Elective surgery of total knee arthroplasty (TKA) is recommended for patients with moderate to severe pain who do not respond to symptomatic therapy [8]. The benefits of TKA are dramatic and include restoring the function and the quality of life for those with knee pain [9-12]. However, since the procedure is elective, help seeking behaviour and several other factors play an important role in utilization of this surgical procedure. Studies have shown that one third of knee replacement candidates in the UK will not accept the surgery if offered to them [13] and out of those on the waiting list for TKA, 25% ended up not having their joint replaced [14]. In North America it has been shown that fewer than 10% of patients who are appropriate candidates for arthroplasty are willing to undergo this procedure [3,15]. Factors such as patient preferences [15], perceptions of the efficacy of the procedure [16,17], patient beliefs [18], expectations of surgery [19,20], TKA experiences of other people [21] and coping mechanism [18] in addition to the physician’s opinion of the procedure [22] all have a major impact on utilization of TKA. These factors will lead to patients either not having the surgery or delaying the procedure unnecessarily until the outcome of surgery becomes less optimal [9,23]. Review of the literature indicates that these factors remained important determinants of undertaking TKA even after adjusting for other socio-economic or demographic factors such as ethnicity, gender, geography and access to healthcare [3,24-26]. The literature also suggested that female patients with OA underutilize TKA when compared to male patients; often not receiving the surgery until they are at a more advanced stage of the disease [3,27].

TKA has been offered to patients in many countries in the Middle East and the Gulf region without adequate understanding of patients’ perceptions and preferences, and thus health care professionals may lack the contextual perspective necessary to identify what implications of disease and treatment outcomes are most important to patients with knee OA. Exploring pain experiences of patients with knee OA, as well as their perceptions and expectations of TKA would potentially improve the ability of health care professionals to provide effective and relevant care for the growing number of patients with knee pain in the Middle East and Gulf region. We aimed to conduct focus group discussions to study the pain experience and mobility limitation of patients as well as to investigate patient decision making to undertake TKA among women with knee pain in the waiting list for surgery. It was anticipated that this methodology would generate revealing comments and insights about the decision-making process as well as the social contexts in which patients make decisions, which are critical information to improve the outcome of the surgery.

## Methods

The study was conducted in Kuwait, a small-country in the Gulf region with a population of 3.5 million, two thirds of whom are non-Kuwaitis. Public health services are easily accessible particularly for Kuwaitis. There is one public orthopaedic hospital (Al-Razi hospital) that performs TKA with approximately 120 surgeries per year but there is also unknown numbers of TKA that are conducted abroad and small number in the private sector.

Five focus group discussions were conducted in the orthopaedic hospital with female patients selected from the waiting list for TKA. Discussions were conducted sequentially in the hospital and were led by a local female facilitator who is a clinical psychologist and proficient in both English and the local Arabic dialect. The note taker was also a local Kuwaiti female. It was important to have a gender-matched facilitator since older women in this conservative society are unlikely to share their experiences in the presence of males. The group discussions were focused on the pain experience of patients and its impact on their social, emotional and physical lives as well as on the factors considered to be important in the decision-making process to undergo TKA. A question guide including eighteen questions with probes were developed in Arabic for use in the group discussions but one question was removed after the first group discussion due to redundancy (See the list below). The groups discussed the impact of knee OA in broad terms and then turned to the process of decision-making about the surgery.

Q**uestion guide used in focus group discussions**

Elements of decision making /Perception of risk of surgery (Risk of complications/fear of surgery)

Q13. Who suggested knee replacement as a treatment for your knee-pain?

Probes: Doctor (at primary care, specialist), friend, family members?

Q14. Do you know anyone who has had knee replacement surgery?

Probes: if so, how has talking with that person made you feel about knee-replacement?

Q15. Other than your doctor, who else did you talk (consult) with on whether to undergo the surgery?

Q16. What would you need to hear from your doctor that would make you agree to undergo a Knee replacement?

Q17. When do you think a person with knee pain should decide to undergo knee replacement surgery?

Knowledge and expectation of Knee arthroplasty

Q8: What can clinicians/doctors do to help you with your knee pain?

Q9: How successful do you think knee replacement is in terms of pain relief?

Q10: How successful do you think knee replacement in terms of improved function/mobility?

Q11: How long do you believe the prosthesis will last for?

Q12: Where did you get your information about knee replacement?

Living with knee pain/ impact of knee pain on patients and their relatives

Q1: How do you feel about your knee pain?

Q2: How has having knee pain interfered with your life?

Probes: social, emotional, and physical?

Q3: From what we have discussed so far, what bothers you the most about having knee pain?

Q4: Is any member of your family affected by you having knee pain?

Probes: If yes, who and how?

Q5: What do you personally do to deal with your knee pain?

Q6: How do you cope with your knee pain?

Q7: Who do you think can help you with the knee pain?

All focus group discussions were audio-recorded and transcribed verbatim using the audio-records and supplemented by the notes. The transcripts were checked against the audio-tapes for completeness and accuracy by another person. Each transcript was coded for themes by two researchers (AT and RS) who then met to compare themes and their organization, working out a set of final themes and subthemes by consensus. Analysis began with identification of key themes and patterns from the data using the process of coding that involved assigning labels to the different themes that emerged from each question. Transcripts were coded by assigning labels to segments of text in themes and then writing descriptive accounts based on these themes [28]. Coding was also checked by another researcher. A final reading of all transcripts was done to confirm the validity of the themes and conclusions. Qualitative data analysis was conducted on the original Arabic transcripts with no translation to any other language. In order to present our findings in this article, translations of the quotes and question guide was done by two independent researchers fluent in both Arabic and English.

Before the start of each focus group discussion, permission to tape-record the interview was taken and a written informed consent was obtained from each participant. The study was approved by the ethics committee at The Faculty of Medicine, Kuwait University.

## Results

A total of 39 patients participated in the group discussions; with a mean age of 62.5 (Standard Deviation, 7.9) years. The demographic characteristics of the participants in group discussions are illustrated in Table 1. One third of the participants had no formal education and another third had primary or intermediate school education. The total duration of group discussions was 289 minutes and the amount of transcripts exceeds 87 pages. The discussions centered around 6 themes that are illustrated in Table 2. Below is a description of participants' responses by theme.

**Table 1:** Socio-demographic characteristics of 39 women with severe knee pain participated in focus group discussions

**Socio-demographic characteristics**		
**Age, mean (SD)**		**62.5 (7.9)**
		**n(%)**
Marital status	Married	26(66.7%)
	Widowed/Divorced	12(30.7%)
	Single	1(2.6%)
Level of education	No formal education	13(33.3)
	Primary/intermediate school	13(33.3)
	High school	7(17.9)
	University degree or above	6(15.4)
	Housewife	27(69.2)
Occupation	Retired	7(17.9)
	Working	4(10.2)
	Housewife	28(71.8)

**Table 2:** Summary of themes and sub-themes generated from focus group discussions

**Theme**		**Patients from waiting list of (orthopaedic hospital)**		
		**Number of times**	**Number of participants**	**Number of groups**
1) Pain-experience and impact of knee pain on patients	Intensity/severity	49	23	5
	Frequency and duration	13	13	5
	Interference with sleep	16	14	5
	Effect of pain on mood	29	14	5
	Management of knee pain	50	27	5
2) Mobility limitations and the need for assistance	Going to bathroom/daily life activities	63	27	5
	Missing leisure/social activities	26	19	4
	Fear of falling	10	8	5
	Difficulty praying normally	7	12	4
	Problem in doing household chores	15	9	4
3) Effect of participants’ pain and or immobility on family members	Family/children are not going out	29	14	5
	The work or the study of sons or daughters are affected	8	6	4
4) Past-medical treatment	Consultation abroad	12	9	4
	Describing past-treatment as a waste of time and money	48	24	5
5) Decision-making to undertake knee arthroplasty and pathway of care	Discussion with family member	37	30	5
	Know someone had knee arthroplasty	32	23	5
	Professionals medical advice to undergo knee arthroplasty	39	27	5
	Fear of surgery	30	26	5
6) Expectation of knee arthroplasty	Improvement of pain	7	11	4
	Improvement of physical functionality	17	12	4
	Life-expectancy of the prosthesis > 10 years	13	12	4

### Pain-experience and impact of knee pain on patients

Pain was a predominant feature of daily living and many participants talked at length about their pain experiences. Participants described their knee pain more than any other issue using such strong expressions as “horrible” or “a complete destruction”. One participant noted, “Horrible pain, you cannot imagine it, I mean unbearable I live only on painkillers which started to hurt my stomach but without these painkillers I just cannot live.” (77-years-old woman).

The use of painkillers and other methods of pain relief were mentioned in every group discussion, most of the time linked with the participants’ fear of the side effects of these analgesics on their stomach, blood pressure, and kidneys, or even fear from becoming dependent on the analgesics. Participants described all kinds of pain management from using tablets and injecting medicines into their knees to acupuncture, and electrical therapy or seeking help from traditional healers. The duration of knee pain was very long for many patients who reported having pain for more than 20 years. Many participants described how pain has interfered with their sleep (14 participants in five groups) and that they must take large amounts of painkillers in order to have a restful sleep. The psychological impact of knee pain was obvious among participants many of whom started crying when they described their pain experiences (six participants in four groups). Some participants described how they turned to religion and reading Holy Quran. They asked God for help, and when they felt hopeless about improvements in their pain asked God to take their lives. Some participants felt more reassured explaining their belief that God would reward them because of their suffering. Participants also described how they distract their attention away from their pain by talking with relatives over the phone or requesting the company of their grandchildren. As one participants said,

“I ask my grandsons to come, I call my daughters and ask them to bring their kids, I try to distract my attention from pain with them. The daughter of my son is 2 month old I want her to be beside me so that I forget the pain.” (48-years-old woman)

Of note is that most of the participants viewed knee pain as an illness that has its causes about which they asked the moderator. They talked about possible causative factors such as obesity (two participants in one group), food (one participant in one group), sedentary life style (one participant in one group), genetics (one participant in one group) or even evil eye (two participants in one group). Only one participant cited that her pain has increased with age but none described their knee pain as part of normal ageing.

### Mobility limitations and the need for assistance

Pain was strongly related to mobility restriction, which was a major theme in all group discussions. Mobility restriction was severe among these groups of patients, has lasted for a long period, and affected their daily activities and social life such as attending social events like weddings. Some participants have not climbed a flight of stairs at their homes for more than 10 years and many were unable to go to the bathroom alone. Fear of falling was also common among participants (eight participants in five groups) who refrained from movement because they had experienced falling several times. Although many participants have domestic helpers, inability to do the household chores was a concern for several participants (nine participants in four groups).

The experience of mobility restriction was shaped by their feeling about their roles as housewives or mothers who are expected to take care of the family. One participant noted while she was crying, “I have a 19-year old disabled son whom I used to take care of; now I sit helpless beside him; now we are both disabled”. Another participant was in sorrow as her two disabled children stay at home because she is physically unable to take them out. Another participant was not happy because her husband started helping her and she is not able to pick her children up from school. Despite their pain and mobility limitation, participants continued serving their husbands and taking care of their children but felt helpless or less valuable when describing their failure to fulfill their felt obligations towards their husbands or children; with two participants expressed their acceptance for their husbands to remarry. The other activity, which participants valued most, was praying which all participants continued to perform albeit sitting down on a chair.

### Impact of pain and/or mobility limitation upon family

Family was an important element in both pain experience and mobility limitation of patients. When participants were asked about persons who help them whenever they experience knee pain, all participants mentioned their sons, daughters or husbands. Participants described in detail how their daughters and sometimes husbands persistently offer help. Participants also described how their sons and daughters or even grandchildren react every time they saw them suffering from knee pain. Sons and daughters bring them everything they need without request and grandchildren urge their parents to take the participants to the doctor. Some participants reported that their sons and daughters do not go to work when the participants have a lot of pain and another participant described how her daughter withdrew from her university course so as to be available for her care. Two participants talked at length about the support they received from their husbands while another participant was in sorrow because her husband let her down and did not support her. Furthermore, social life and leisure activities of the whole family were affected because families opted against going out and leaving the participants at home alone. Participants described how their young children and teenagers cannot go out without supervision and thus had to stay at home most of the time; with girls who prefer to go out with their mothers, are particularly affected. What can be drawn from this is that pain and mobility limitation experienced by participants affected their families; and that family support was a critical factor in the coping with the pain.

### Past-medical treatment

Participants brought up their past-medical treatment several times during the discussions. They mentioned all different kinds of pain management from tablets to acupuncture. Some participants described taking expensive injections. Participants judged their past-medical treatment as an overuse of painkillers and pain management procedures and as a waste of time and money. Pathways of care in this group of patients also included consultation in neighboring countries including Bahrain, Syria, Dubai, Saudi Arabia, Jordan and other countries. Nine participants in four groups talked about at least one consultation abroad. Most of these consultations occurred when patients accompanied their families for holiday abroad; and resulted in receiving recommendation for TKA but none of the patients followed the recommendation at that time. As one participant said “I have only one son who took me almost to every country. He took me to Syria, Bahrain, Jordan, German Hospital in Jeddah and wanted to take me to Dubai to have the operation done”.

### Process of decision making to undergo TKA and pathway of care

Pathway of care showed that participants had received the advice to undertake TKA at least once locally and once abroad but this was very late and came after a long and unnecessary suffering from their perspective. There was consensus that the medical advice to undertake TKA was very late. One participant said "they wasted our life, they should have told us to have the operation from the beginning" while another noted “doctor do not advise their patients to undertake the operation until the knee is completely destroyed then they give their advice”. More illustrative quotes on the doctors’ advice are shown below. Part of the delay in medical advice to have TKA came from the fact that patients sought second, third and probably fourth opinion on their OA from different clinicians who failed to consider the treatment which patients had received from previous clinicians and thus started another trial of painkillers; as one of the patients put it “every doctor wants to try from the beginning to see if we need surgery”.

Although there was delay in clinicians’ recommendation for surgery, further and even longer delay was caused by the participants’ deliberation on whether to undergo the operation. Factors that caused this delay are interlinked and include: a) Fear of the operation b) Seeking the encouragement and approval of family members on whether to have the operation, and if so, abroad or locally c) Lack of information about the procedure and the outcome d) Frequent consultations whereby every clinician tried medical treatment without considering the outcome of past medical treatment.

Participants’ fear of the operation was a strong reason for delaying the operation even after they received the clinical advice for arthroplasty. Twenty-six participants in five groups have mentioned fear of the operation which was either related to fear of the operation itself, the anesthesia, pain after the surgery or fear that the operation may not produce the hoped outcome. One participant described how her excessive fear of undergoing a previous surgery to remove a tumor caused her blood pressure to be extremely high and as a result, was sent home without having the surgery. Fear of the operation was exacerbated by the fact that some clinicians advised against knee arthroplasty because they wanted patients to exhaust all possible non-invasive medical treatments (See quotes below). As a result, patients remained anxious and doubtful about the surgery, even though clinicians changed their minds and recommended the surgery when patients did not respond to the medical treatment.

Facilitator: Is there anything that doctors can do to help?

W1: They do not recommend the operation! They don’t recommend the operation! Straightaway physiotherapy, go and do electrical massage or injections; can you imagine I feel I am dying.

W2: Four years ago I consulted a private doctor, first he advised me not to have the operation and to be away from it, my family wanted to take me to Germany ….he advised me not to have the operation even if I have to walk on cane stick when he realized there is no hope he said now you have nothing other than the operation!

W1: For 20 years he has not recommended the operation!

W3: For 30 years he has not recommended the operation!

W4: No he advised me to have the operation from the beginning, the visiting doctor said it is necessary

Quotes from another focus group:

W1: Doctor does not recommend the operation, he does not say straightaway to the operation!

W2: Yes, he does not say straightaway to the operation!

W3: We recommend this, they should recommend the operation before other disease comes

W1: Yes. . .yes

Facilitator: Do you agree …

W3: Exactly

W1: Yes right

W4: wait until our life is over!

When the participants were asked about those with whom they had discussed the possibility of having TKA, the majority of the participants mentioned their sons, daughters and husbands. One participant said her sons were not happy that she was going to have the operation because they believed she is too old. Another participant said that initially her husband was against the operation asking her to wait until new advancements in medicine occur but changed his mind when he saw the successful outcome among those who had TKA. There was a long debate within the participants’ families on whether to have the surgery at all and, if so, whether to have it done locally or abroad.

Many participants knew someone who had TKA and some of whom were their own relatives (23 participants in five groups). People who had the operation were mentioned as a source of information and their experience was brought up several times. All participants cited positive experiences of people who had undergone the operation. A participant described a success story of a woman who, after the operation, is now able to climb the stairs to reach her flat on the fifth floor while another participant cited a story of a woman who is able to walk around the Ka'aba in Mecca (i.e. some of the steps involved in the Islamic Pilgrimage to Mecca). Many others cited positive and encouraging experiences of other patients. Of note is a participant who was advised against having TKA from a lady in a wheelchair who had the prosthesis that failed after 20 years. Instead of being discouraged, the participant became more positive about having the surgery because of her new understanding that the prosthesis lasted for 20 years. Knowing someone who had knee arthroplasty increased the knowledge of the participants about the procedure and raised their expectations about the positive outcome of the surgery.

As mentioned above, there was a delay of years between the clinical recommendation for TKA and the decision to undertake the procedure because patients were ambivalent about TKA. Participants attributed this indecisiveness to the poor quality of clinical advice, which did not include explanations of the expected outcome in terms of pain relief, improved mobility or the life-expectancy of the prosthesis. Some participants noted a difference between private and public sector doctors in the way they explain treatment options to patients. They explained how clinicians in the public sector simply ask "do you want the surgery or not", and do not provide any written or verbal information about the surgery. Most participants gleaned their information on TKA from those who had previously experienced it and this took time to occur and caused substantial delay. Participants on the waiting list had many unanswered questions about their upcoming surgery and sought the answers from the moderator. It was surprising to find that participants expressed full trust in their surgeons but at the same time expressed a strong sense of dissatisfaction with the insufficient amount of information provided by their surgeons. One participant said “you have to ask and persist in order to get any piece of information”.

### Expectations of total knee arthroplasty

When the participants were asked about their expectations from the surgery, their answers ranged from “Do not know” to “God knows”, or citing the positive experiences of those who had TKA. The experience of those who had TKA appeared to improve the participants' expectations of surgery. As mentioned above one participant knew a patient who is able to climb stairs and another knew someone who went to Mecca and were able to complete their religious practices. Other participants had modest expectations from surgery such as walking short distances without pain or going to the bathroom alone. One participant had asked the surgeon about whether she can pray-which requires kneeling- after the operation without siting on a chair. Overall, lack of information has contributed to poor and non-specific expectations of TKA among the participants.

## Discussion

This is the first qualitative study that has attempted to describe the pain experiences of women with advanced OA and their expectation of TKA in the Middle East or the Gulf Region. Given the ageing population and the increasing prevalence of obesity in the area, the morbidity associated with knee osteoarthritis is expected to increase. It has also been suggested that women delay seeking arthroplasty until a later point in the process of functional loss [27,29]. Our findings showed that family was at the centre of patients’ pain experience and mobility limitation both of which affected patients’ families. Additionally, there was an obvious involvement of the family in the pathway of care and the decision to undergo TKA. Consultation abroad seems to be part of the pathway of care in this group of patients and this has an implication on patient’s decision to undertake TKA. Pathway of care highlighted two types of delay; the first was due to late medical advice to undertake TKA; and the second occurred after the medical advice for TKA and was attributed to patients’ ambivalence regarding undergoing arthroplasty.

Unlike the underlying cultural concept of ageing in the Western societies, which accepts knee pain as an inevitable part of the normal ageing process that does not require professional medical treatment [30], our participants view knee pain as a medical condition that has its own causative factors such as obesity, food, sedentary life style, genetics or even evil eye. Our participants did not attribute their knee pain to their age although their age was comparable to patients in Western societies and also have the same pattern of co-morbidities in older age [31,32]. This may have suppressed the acknowledgment that some pain should be tolerated and thus explains the numerous consultations and treatments that were discussed. This is different from that reported elsewhere where women with knee or hip pain wanted to be stoic and did not want to bother the doctor or take the medications [33] or thought nothing could be done to improve their condition [34].

Patients described their pain experiences showing that pain has interfered with their sleep which is consistent with previous studies that showed sleep disturbance is common among patients with OA [35,36]. It has also been suggested that poor sleep exacerbates pain in people with chronic pain disorders like OA, by decreasing pain tolerance [37,38], although the relationship between pain and sleep disturbance remains unclear among patients with OA because both are common in elderly people [35]. The impact of pain on the mood of our participants was obvious as many of them expressed frustration and disappointment and some cried while describing their experiences with knee pain. We support authors who call for strategies to increase the detection and management of mood disorders among patients with OA [33]. Our participants described various methods of coping with pain including turning to religion, calling relatives and taking care of their grandchildren. The later has been described to be a source of Joy for patients in other settings [39].

In the current study, patients’ pain experiences and mobility limitation was shaped by strongly felt obligation towards their families. Participants felt less valuable or helpless when they thought they failed at their obligations towards their husbands or children. Like other studies that described the desire of patients with knee OA to carry on with valued activities even with the presence of pain [39], participants continued taking care of their families whilst experiencing pain. As an example, participants tried to do house chores even with availability of domestic helpers. Clinicians must acknowledge that pain experiences are related to mobility limitations which in turn are related to the social context; and thus simply providing temporary relief for the uncomfortable feeling will not necessarily address the important complications of knee pain. The strong desire of women to fulfill caring responsibilities seems to be a key deciding factor to undertake the TKA.

The pathway of care showed the presence of two major delays with distinct causes before the participants were able to reach a final decision to undergo TKA. The first delay was caused by late medical advice to undertake TKA. There was a consensus that the medical advice was late and came after a long time of unnecessary suffering. Participants felt that the threshold of pain and loss of function at which the knee surgery should be done- described sometimes as breakpoint experience [40]- had been reached a long time before they received the clinical advice for surgery. Patients went through a sequence of clinical consultations seeking second and third opinions from different clinicians each trying conservative treatments and painkillers for a long time before finally advising patients to undergo TKA. In order to convince patient to go through another trial of medical treatment, some clinicians expressed a negative opinion about TKA which had a negative impact on the participants’ decision to have knee surgery even after clinicians later changed their opinion. Doctor opinion has been suggested to have a strong impact on decision to go to surgery [21,22]. Most of the participants sought medical consultations abroad which usually occurred when patients accompanied their families during summer vacation. Consultation abroad seems to be an important part of the pathway of care for the patient with chronic knee pain in Kuwait.

The second delay came after the medical advice to undergo TKA due to the patients’ extended period of indecisiveness regarding the procedure. Patients attributed this lengthy time of thinking to the poor quality of advice that was not accompanied by explanation about the procedure and its outcome. Fear of the operation itself and fear of poor outcome which has been described to be common among women [33], has contributed to this delay. Lack of information about TKA extended this delay as patients had to wait for their information about TKA to accumulate and sought advice of those who had TKA. Similar to other settings [28], participants expressed a strong desire for information about the surgery and what to expect. Most participants knew someone who had the procedure and cited positive experiences that improved their expectation of TKA and helped them reach a favourable decision. In other settings, the experience of others who had a joint replacement influenced the choice of individuals to have or not to have joint replacement [21,41]. After the medical recommendation to have TKA, there was a long debate within the family on whether to have the surgery at all and, if so, locally or abroad. Similar to other settings, conflicts of opinion between the patients and family members hampered satisfactory decision making [28] but unlike other settings where patients were only interested to know what their families thought of the surgery[40], in our settings, family members were involved in all steps of pathway of care.

Doctor competency or doctor trust was not an issue in deciding to undergo TKA although this may be because of selection bias as all participants were from the waiting list for TKA. Despite the complete trust in their surgeons, participants expressed dissatisfaction with the insufficient amount of information they were given or that they were not listened to. Women elsewhere have emphasized good listening as a quality of a good doctor [33]. Unlike other settings [33], participants did not show concern about acute postoperative care or having no one to care for them nor concern about the healthcare cost because patients are entitled to have the procedure for free.

The purpose of focus group discussions is usually to generate revealing comments and insights about a topic in a spontaneous but focused conversation among strangers. Our findings cannot be generalized to all patients with knee pain in Kuwait because the study focused on a relatively homogenous group of Kuwaiti female patients living with advanced OA recruited from a specialized orthopedic hospital. They cannot also be generalized to patients with early symptoms of knee OA or those who seek TKA in private hospitals or abroad.

## Conclusion

Our findings emphasize the need for improved doctor-patient interactions and highlighted several factors in the pathway of care of patients with chronic knee pain including seeking multiple clinical opinions both locally and abroad and the lack of patient education, which is a significant part of preoperative rehabilitation. The provision of both verbal and written information around TKA seems to be critical to improve patient understanding of the risks and benefits of TKA and allows patients to make an informed decision about the procedure and thus achieve patient-centered healthcare.

## Competing interests

The authors declare that they have no competing interests.

## Authors’ contributions

AT: Designed the study, wrote the grant protocol, analyzed the data and drafted the paper. RS: Contributed to the study design, data collection, data analysis and revised the manuscript for important intellectual content. ES: Contributed to data collection, data collection, data analysis and revised the manuscript for important intellectual content. DS: Contributed to data collection, data collection, data analysis and revised the manuscript for important intellectual content. SM: Contributed to data collection, data collection, data analysis and revised the manuscript for important intellectual content. All authors read and approved the final manuscript.
